# Expanded *in vivo* substrate profile of the yeast N-terminal acetyltransferase NatC

**DOI:** 10.1016/j.jbc.2022.102824

**Published:** 2022-12-22

**Authors:** Petra Van Damme, Camilla Osberg, Veronique Jonckheere, Nina Glomnes, Kris Gevaert, Thomas Arnesen, Henriette Aksnes

**Affiliations:** 1 iRIP Unit, Laboratory of Microbiology, Department of Biochemistry and Microbiology, Ghent University, Ghent, Belgium; 2 Department of Biomedicine, University of Bergen, Bergen, Norway; 3 Department of Clinical Science, University of Bergen, Bergen, Norway; 4 VIB-UGent Center for Medical Biotechnology, Ghent, Belgium; 5 Department of Biomolecular Medicine, Ghent University, Ghent, Belgium; 6 Department of Biological Sciences, University of Bergen, Bergen, Norway; 7 Department of Surgery, Haukeland University Hospital, Bergen, Norway

**Keywords:** N-terminal acetylation, glycerol metabolism, mitochondrial metabolism, N-alpha-acetyltransferase 30, non-fermentable sugar phenotype, N-terminal acetylome, protein modification, subcellular fractionation, *S. cerevisiae* disease model, virus assembly, GNAT, GCN5 (General Control Non-repressed protein 5)-related N-acetyltransferase, Mak, maintenance of killer, NAA30, N^α^-acetyltransferase 30, NAT, N-terminal acetyltransferase, *Nfs*^*–*^, non-fermentable sugar phenotype, Nt-Ac(etylation), N-terminal acetylation

## Abstract

N-terminal acetylation is a conserved protein modification among eukaryotes. The yeast *Saccharomyces cerevisiae* is a valuable model system for studying this modification. The bulk of protein N-terminal acetylation in *S. cerevisiae* is catalyzed by the N-terminal acetyltransferases NatA, NatB, and NatC. Thus far, proteome-wide identification of the *in vivo* protein substrates of yeast NatA and NatB has been performed by N-terminomics. Here, we used *S. cerevisiae* deleted for the NatC catalytic subunit Naa30 and identified 57 yeast NatC substrates by N-terminal combined fractional diagonal chromatography analysis. Interestingly, in addition to the canonical N-termini starting with ML, MI, MF, and MW, yeast NatC substrates also included MY, MK, MM, MA, MV, and MS. However, for some of these substrate types, such as MY, MK, MV, and MS, we also uncovered (residual) non-NatC NAT activity, most likely due to the previously established redundancy between yeast NatC and NatE/Naa50. Thus, we have revealed a complex interplay between different NATs in targeting methionine-starting N-termini in yeast. Furthermore, our results showed that ectopic expression of human NAA30 rescued known NatC phenotypes in *naa30*Δ yeast, as well as partially restored the yeast NatC Nt-acetylome. Thus, we demonstrate an evolutionary conservation of NatC from yeast to human thereby underpinning future disease models to study pathogenic *NAA30* variants. Overall, this work offers increased biochemical and functional insights into NatC-mediated N-terminal acetylation and provides a basis for future work to pinpoint the specific molecular mechanisms that link the lack of NatC-mediated N-terminal acetylation to phenotypes of NatC deletion yeast.

N-terminal acetylation (Nt-acetylation) is a highly conserved protein modification that occurs on the majority of eukaryotic proteins (1, 2, 3, 4, 5). This modification is essential for human health (6, 7, 8, 9, 10, 11, 12) given its importance for several types of protein and cellular regulatory mechanisms such as protein–protein interactions (13, 14, 15, 16, 17), membrane affinity and subcellular targeting (18, 19, 20, 21, 22), protein turnover (23, 24, 25, 26, 27, 28, 29), and processes connected to protein aggregation and folding (30, 31, 32). Increasing evidence suggests Nt-acetylation itself might be under regulatory control (33, 34, 35) and potentially take part in an intricate interplay with other N-terminal modifications (36, 37, 38) or lysine acetylation (39, 40).

The widespread occurrence of protein Nt-acetylation is due to a machinery of N-terminal acetyltransferases (NATs). These enzymes catalyze Nt-acetylation by transferring an acetyl moiety from acetyl coenzyme A to the free α-amino group of protein N-termini. Eight NATs have been defined thus far, of which NatA to NatE are conserved from yeast to humans, whereas NatF (2, 41, 42) is an organelle-bound NAT found in plants and higher eukaryotes, NatG (43) and some other emerging NATs are only found in plants (39), and NatH (44) expression is restricted to animals (4, 40). Overall, Nt-acetylation and the NAT machinery are highly conserved, with the yeast *Saccharomyces cerevisiae* model having contributed significantly to advancing our knowledge on this intriguing modification. In yeast, NatA, NatB, and NatC are considered the major NATs estimated to Nt-acetylate up to 31%, 17%, and 10% of all yeast proteins, respectively (45). Yeast NatA (yNatA) acts on N-termini starting with S, A, T, G, C, or V (1, 46, 47) residues that are typically exposed upon removal of the initiator methionine (iMet) by methionine aminopeptidases (48). yNatB acetylates ME, MD, MN, and MQ N-termini (47, 49, 50), while yNatC was found to acetylate some iMet-starting hydrophobic N-termini, ML, MF, MI, and MW (47, 51, 52, 53, 54). Although the major determinants for substrate specificities of these major *S. cerevisiae* NATs have been defined, proteome-wide *in vivo* substrate profiles have thus far only been reported for yNatA (1) and yNatB (50).

NatC is a NAT complex consisting of three proteins, Naa30, Naa35, and Naa38 (also referred to as Mak3, Mak10, and Mak31, respectively) (53). These proteins were named MAK (maintenance of killer) because they were initially found to be among the 29 chromosomal genes required for the propagation of the killer protein toxin M1 encoded by the L-A dsDNA virus in yeast (55, 56, 57). Naa30 is the subunit containing the catalytically active GNAT (GCN5 (General Control Non-repressed protein 5)-related N-acetyltransferase) fold, whereas Naa35 and Naa38 are auxiliary subunits required for NatC activity (53). The human NatC complex was found to consist of orthologs of all three yeast NatC subunits (58). Importantly, Polevoda and Sherman (53) showed that yeast needs all three subunits for yNatC–mediated acetylation. Thus, ablation of any of these subunits is thus referred to as NatC-deficient yeast. Further evidence for the existence of a trimeric complex is that several *naa30*Δ, *naa35*Δ, and *naa38*Δ phenotypes are shared. All genes are necessary for L-A virus propagation (55, 56, 57) due to NatC-mediated Nt-acetylation of the L-A *gag* protein which is crucial for viral particle assembly and thus indirectly also for viral M1 toxin production (51). Moreover, osmotic sensitivity was revealed by reduced growth ability under high salt stress (1M NaCl) for all NatC subunit mutants (53). Recently, the structures of the heterotrimeric *S. cerevisiae* and *S*. *pombe* Naa30–Naa35–Naa38 complexes were solved (59, 60). These structures revealed the structural basis for the dependency on all three subunits for normal NatC activity and showed a catalytic cleft suitable for accommodating hydrophobic N-termini as well as an overall structure differing somewhat from NatA and NatB complexes (61, 62).

One of the reported phenotypes of NatC-deficient yeast is that certain NatC substrates lose their subcellular localization: the Golgi proteins Arl3 (18, 19, 63) and Grh1 (64) mislocalize to the cytoplasm, whereas the inner nuclear membrane protein Trm1-II shifts to a nucleoplasmic localization (63, 65). The ability of NatC to aid the correct subcellular localization of its substrates has thus been suggested. However, a small-scale screen argues against such generalization of NatC-dependent substrate localizations as none of the 13 NatC substrate candidates tested changed localization in the absence of Naa30 (63). Nevertheless, known localization-dependent cases, like Arl3, may conveniently be used as a functional readout of NatC activity *in vivo* (66).

Individual deletion of any of the three NatC subunits results in reduced growth in non-fermentable sugars referred to as the *Nfs* ^*–*^ phenotype (53), indicative of a diminished ability to utilize non-fermentable carbon sources. Another, perhaps related finding, is that *NAA35* expression is glucose-repressible and highly elevated when yeast is grown on glycerol compared to growth on dextrose (67). Furthermore, a large-scale screen for oxygen response mutants found a 29% reduction in colony size of the *naa30*Δ mutant under anoxic growth conditions (68). Considering these *Nfs* ^*–*^ growth and respiratory effects, it is possible that NatC is responsible for acetylating one or several proteins that are directly or indirectly involved in anoxic growth. As glycerol is metabolized through a different pathway than glucose (69, 70, 71), proteins involved in this pathway or in the glycerol-responsive induction of them could thus possibly depend on NatC-mediated Nt-acetylation. Growth on glycerol and other non-fermentable carbon sources prompts yeast to rely on oxidative phosphorylation and proper mitochondrial function. An extensive mitochondrial volume is also observed in yeast growing in glycerol medium (72). Growth defects on glycerol could therefore reflect a mitochondrial disturbance that is caused by lack of Nt-acetylation of proteins important for mitochondrial function. The mitochondrial proteins Kdg1, Fum1, and Mrp1, whose null mutants cause *Nfs* ^*–*^ have all been suggested as putative NatC substrates due to their N-terminal P1-P4 sequence being MLRF, same as for L-A *gag* (54), although this has never been experimentally confirmed. In human cells, NAA30 depletion has been reported to reduce the expression levels of mitochondrial proteins and to result in mitochondrial fragmentation and loss of mitochondrial membrane potential (73).

Deletion of y*NAA30* has also been reported to cause abnormal elongation of a specific structure found in the yeast plasma membrane called furrow-like invagination or MCC (membrane compartment of Can1) patches (74). In contrast to NatA and NatB (47, 75), deletion of the NatC catalytic subunit does not affect mating efficiency in yeast (53). Starvation-induced nuclear-to-cytosolic relocalization of the proteasome, an age-dependent process, was dependent on Nt-acetylation by NatB and NatC, but not NatA (76). This work also showed that loss of NatA or NatB compromised proliferation capacity in starved cells, whereas loss of NatC specifically affected growth of replicative old cells.

Various NAT deletion phenotypes are likely manifested through defects of their substrates suffering a lack of Nt-acetylation. In this respect, the consequences resulting from Nt-acetylation gained interest in recent years and several aspects of protein functionality have been shown to be affected by the lack of Nt-acetylation. Very few reports connect NAT deletion or knockdown phenotypes to specific functional impairments of particular NAT substrates. For NatC, it is thus far unknown through which substrate(s) the yeast *Nfs* ^*–*^ phenotype manifests itself. Thus, the identification of substrates and detailed characterization of NatC’s Nt-acetylome may provide a first understanding of the functional proteome-wide implication of protein Nt-acetylation by NatC.

In this study, we performed N-terminal proteomics on (subcellular) proteome extracts of *naa30*Δ and WT yeast to comprehensively identify *in vivo* yNatC substrates and determine the substrate profile of *S. cerevisiae* NatC in more detail. A *naa30*Δ strain expressing the human ortholog was analyzed alongside to investigate the evolutionary conservation of NatC substrate specificity between yeast and human. Furthermore, we utilized the previously characterized *naa30*Δ phenotypes, Arl3 mislocalization, and *Nfs* ^*–*^, to probe for the ability of human NAA30 to functionally compensate for the lack of Naa30 in yeast.

## Results

### Human NAA30 rescues the *naa30*Δ Arl3 mislocalization and *Nfs*^*–*^ phenotypes

**Figure 1:**
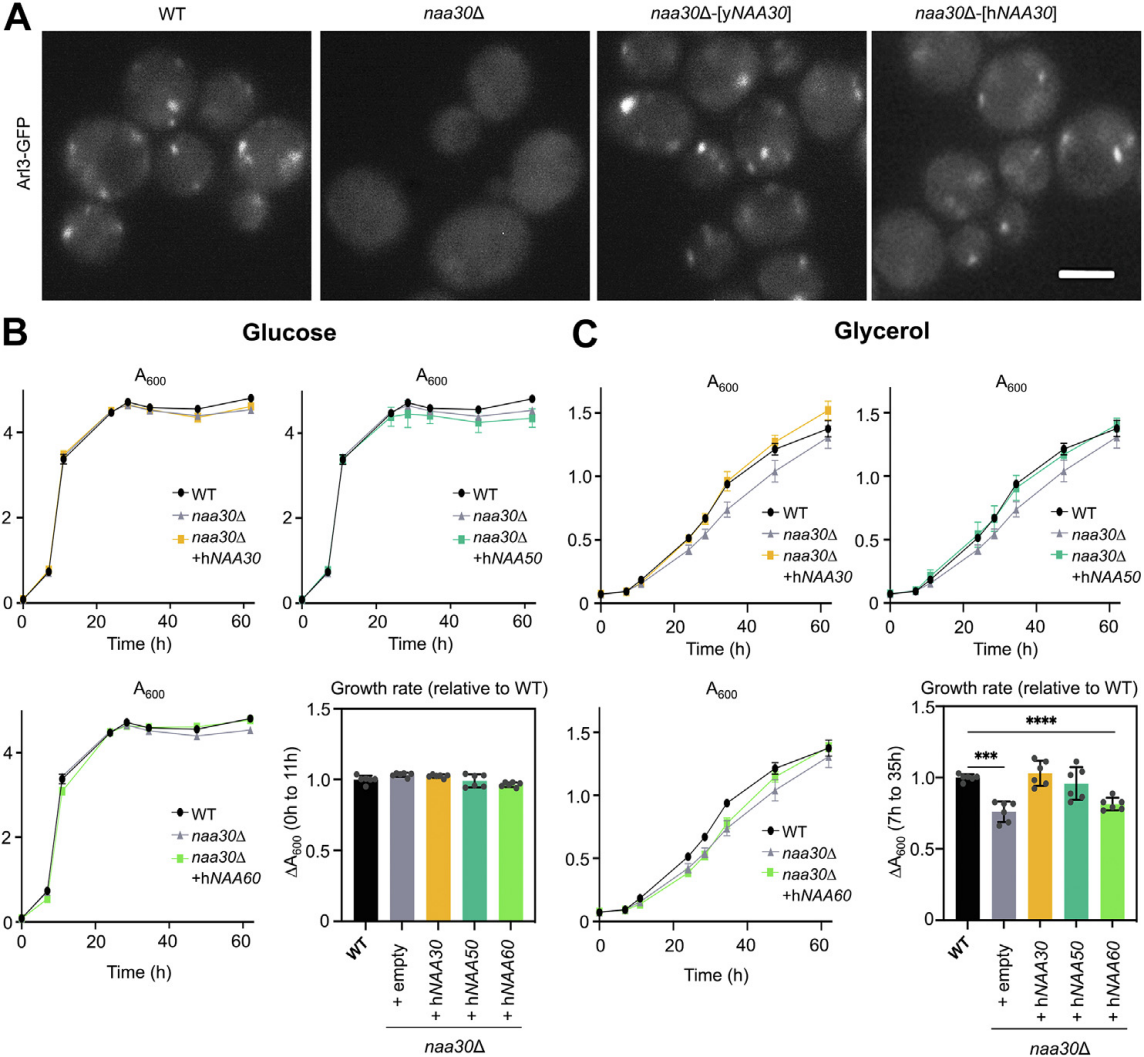
**Human NAA30 rescues Arl3 localization and *Nfs***^–^**phenotypes of *naa30Δ* yeast.***A*, live imaged Arl3-GFP yeast of the indicated genotypes. Shown are representative zoomed fields (scale bar represents 2.5 μm), based on images from > 100 yeast cells from approximately 20 random fields of view per genotype. *B* and *C*, cells were cultured in medium with either glucose (*B*) or glycerol (*C*) as the sole carbon source and the cell density was measured by optical density (A_600_). The bar charts show the increased cell density, A_600,_ from initial to late exponential growth phase. All data shown represent the mean of three technical replicates from two biological replicates (sister clones) per genotype (n = 6 in total per genotype), and all error bars show SD. Data were analyzed by a two-tailed *t* test with unequal variance. ∗∗∗ indicate *p*-value <0.0005, ∗∗∗∗ indicate *p*-value <0.00005 and other comparisons to WT (+h*NAA50* and +h*NAA60*) were non-significant.

Sequence alignments have shown yeast and human NAA30 to be highly conserved in the GNAT domain (58, 77), thus pointing to the possibility of functional replacement. As mentioned above, one phenotype of *naa30*Δ yeast is that the Golgi protein Arl3 mislocalizes to the cytoplasm due to its non-acetylated N-terminus (18, 19, 63). Here, we show that this aberrant localization of endogenous Arl3 can be reversed by ectopic expression of hNAA30 in the *naa30*Δ background (Fig. 1*A*). As an additional control, yNaa30 expression was reintroduced in the *naa30*Δ strain, demonstrating that both orthologs restored the Golgi localization of Arl3, implying that hNAA30 can Nt-acetylate yArl3.

The *Nfs* ^*–*^ phenotype was initially revealed by observing yeast growth on solid media at elevated temperature (37 °C), where growth was unaffected on glucose plates but reduced on glycerol plates (53). In the present work, we performed a growth assay in liquid medium at normal growth temperature (30 °C), thus comparing the two carbon sources under normal, non-stressed conditions. In glucose medium, there was, as expected, no significant difference between the growth of all strains tested (Fig. 1*B*). When grown in glycerol medium, the *naa30*Δ strain showed a somewhat reduced proliferation, a modest impact compared to the previously reported growth defect observed at elevated temperature (53). During the exponential growth phase where the difference was most prominent, the proliferation was significantly reduced in *naa30*Δ compared to the control strain (Fig. 1*C*). Interestingly, the *Nfs* ^*–*^ phenotype was completely reversed by h*NAA30* expression (Fig. 1*C*), thus suggesting that hNAA30 can functionally replace yNaa30 by Nt-acetylating yNatC substrates besides Arl3. For comparison, we also investigated *naa30*Δ yeast ectopically expressing h*NAA50* and h*NAA60* in parallel, due to their previously reported overlapping specificity in targeting ‘Met-hydrophobic’-type N-termini (2, 78, 79). Previously, ectopic expression of h*NAA60* but not h*NAA50* was shown to rescue the Arl3 localization in *naa30*Δ yeast (66). In the present work, hNAA50 could rescue the glycerol growth phenotype to a similar extent as hNAA30, whereas results for hNAA60 were inconclusive.

### *In vivo* Nt-acetylation activity in yeast expressing y*NAA30*, h*NAA30,* or no *NAA30*

**Figure 2:**
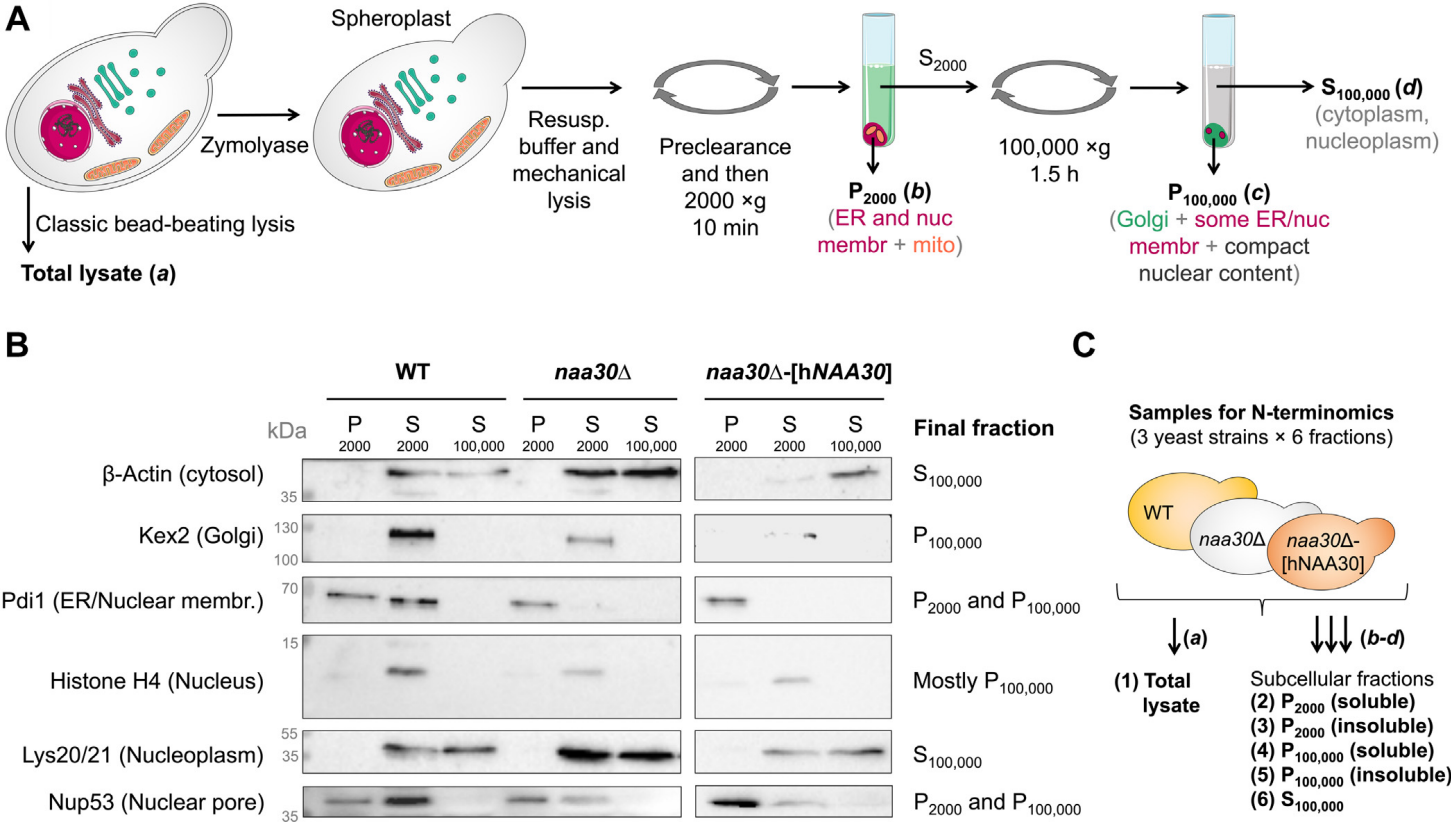
**Preparation and validation of samples for N-terminomics analysis.***A*, yeast extracts were prepared by two methods, classic bead-beating lysis providing a total lysate (*a*) and yeast subcellular fractionation providing three samples (*b-d*) enriched for various cellular compartments. P_2000_/S_2000_: pellet/supernatant after centrifugation at 2000*g*; P_100,000_/S_100,000_: pellet/supernatant after centrifugation at 100,000*g*. *B*, Western blot analysis of yeast subcellular fractions prepared for N-terminomics (COFRADIC). The indicated yeast strains WT, *naa30*Δ, and *naa30*Δ-[h*NAA30*] were subjected to subcellular fractionation as described in panel *A* and Experimental procedures. Aliquots were collected for Western blot inspection and probed using antibodies against the indicated marker proteins. Labels on the *right side* summarize the final segregation of each marker. *C*, in further preparative steps, the pellet samples were divided into soluble and insoluble fractions that were analyzed separately, yielding in total 18 proteome fractions (three different yeast strains and six cellular fractions). COFRADIC, combined fractional diagonal chromatography.

Even though L-A *gag*, Arl3, and Trm1-II are verified yNatC substrates, the Nt-acetylome and overall *in vivo* activity of yNaa30/yNatC remain largely unexplored. We performed N-terminomics analyses on protein extracts from control yeast, *naa30*Δ yeast, and *naa30*Δ expressing hNAA30. To improve proteome/N-terminome coverage, we analyzed both total yeast lysates as well as soluble and insoluble protein fractions obtained upon yeast subcellular fractionation (Fig. 2 and Experimental procedures).

In total, we identified 1596 translation initiation site (TIS) indicative unique N-termini originating from 1444 yeast proteins (Table S1). Of these, 1432 N-termini acquired a start position at amino acid 1 or 2, while 164 started after position 2. The latter category is indicative for the expression of alternative N-terminal proteoforms, for example, resulting from alternative translation initiation (80), as these N-termini were compliant with Nt-acetylation and iMet–processing rules (81, 82) while starting only after protein position 2 (Table S1). As previously observed (1), within the NatA-type substrate category, a large fraction of S, T, and A NatA substrates were Nt-acetylated (98%, 58%, and 60%, respectively), while C, G, and V N-termini were more rarely Nt-acetylated (40%, 13%, and 4%, respectively) (Table S1). Furthermore, as expected, NatB substrates (50) of the MD, ME, and MN types were mostly fully Nt-acetylated (97%, 93%, and 98%, respectively), while MQ N-termini were slightly less Nt-acetylated (74%) (Table S1). Overall, NatA and NatB Nt-acetylation patterns were unaffected when comparing *naa30*Δ *versus* WT yeast, in full agreement with their NAT substrate classification. In general, non-NatB type M-starting N-termini were found more rarely Nt-acetylated (here 40% Nt-acetylated when excluding MP N-termini (non-NAT substrate (81)) vs 94% for NatB substrates) as well as having a lower degree of Nt-acetylation than NatB substrates.

To identify yNatC substrates, we first considered all identified protein N-termini of which the degree of Nt-acetylation could be quantified in both *naa30*Δ *versus* control (WT) yeast N-terminomes (Table S1). By using all fractions, including the total lysate (Fig. 2), we identified a higher number of possible yNatC substrates. We first inspected those cases where yNatC substrates were identified in both soluble and insoluble fractions to check if any notable difference in their Nt-acetylation levels could be observed. Only one such example was found, LKHA4/Lap2, and its shift in Nt-acetylation between insoluble and soluble fraction was modest. All of this indicates that the degree of Nt-acetylation is generally stable across all analyzed fractions.

In total, we identified 57 unique yNatC substrates as partially (5%-95%) or fully Nt-acetylated in the WT strain, while Nt-free (0% Nt-Ac) in *naa30*Δ yeast (Table 1). These substrates covered the previously established yNatC N-terminal substrate specificities comprising ML (21 substrates), MI (9 substrates), and MF (8 substrates) (47, 51, 52, 53), with the exception of MW N-termini (54), as no proteins carrying this less frequent N-terminus type (0.64% of *S. cerevisiae* proteome (2)) were identified. Interestingly, our substrate list also included N-termini starting with MY (5 substrates), MK (7 substrates), MM (4 substrates) as well as MA, MV, and MS (1 substrate of each).

**Table 1:** *Saccharomyces cerevisiae* NatC substrates identified in yeast by means of N-terminomics

Protein (UNIPROT)	Detected Nt-peptide sequence	Peptide P1′	Peptide P2′	Start	Nt-Ac in WT yeast (%)	Predicted NAT type in yeast
HSP77/SSC1/YJR045C	MLAAKNILNR	M	L	1	100	NatC/E type
ODPA/PDA1/YER178W	MLAASFKR			1	97-100	NatC/E type
PRP19/YLL036C	MLCAISGKVPR			1	64	NatC/E type
LST4/YKL176C	MLGNLLR			1	100	NatC/E type
ADRX/YAH1/YPL252C	MLKIVTR			1	100	NatC/E type
BEM1/YBR200W	MLKNFKLSKR			1	100	NatC/E type
NFS1/YCL017C	MLKSTATR			1	100	NatC/E type
CRF1/YDR223W	MLLSAPVNSTVR			1	26	NatC/E type
YG1O/YGR035C	MLLTPAKTTR			1	54	NatC/E type
HUL5/YGL141W	MLNFTGQTR			1	100	NatC/E type
ETR1/YBR026C	MLPTFKR			1	100	NatC/E type
YEY8/YER158C	MLQQGSSSR			1	100	NatC/E type
GSHR/GLR1/YPL091W	MLSATKQTFR			1	100	NatC/E type
NOT1/CDC39/YCR093W	MLSATYR			1	100	NatC/E type
SKS1/YPL026C	MLSDCLLNNFR			1	0-15	NatC/E type
PDC2/YDR081C	MLSIQQR			1	10-15	NatC/E type
BAP2/YBR068C	MLSSEDFGSSGKKETSP…			1	10	NatC/E type
MSN4/YKL062W	MLVFGPNSSFVR			1	100	NatC/E type
AIM18/YHR198C	MLKSLQR	M9	L10	9	80	NatC/E type
LKHA4/LAP2/YNL045W	MLPLSIEQR	M40	L41	40	7-29	NatC/E type
PCD1/YLR151C	MLSSKQLIENLIR	M8	L9	8	58	NatC/E type

AFG1/YEL052W	MIALKPNAVR	M	I	1	100	NatC/E type
YJ133/YJL133C-A	MIAQSTR			1	8-20	NatC/E type
ISF1/YMR081C	MIASEIFER			1	9-13	NatC/E type
HOT13/YKL084W	MIETAIYGKTVDDQSR			1	1-8	NatC/E type
LRG1/YDL240W	MIQNSAGYR			1	100	NatC/E type
SLY41/YOR307C	MIQTQSTAIKR			1	94	NatC/E type
ACM1/YPL267W	MISPSKKR			1	73-94	NatC/E type
SRY1/YKL218C	MIVPTYGDVLDASNR			1	3-8	NatC/E type
TFC6/YDR362C	MIKLR	M246	I247	246	100	NatC/E type

YD239/YDR239C	MFDGFSNNKGKR	M	F	1	100	NatC/E type
RFC2/YJR068W	MFEGFGPNKKR			1	99-100	NatC/E type
RRN11/YML043C	MFEVPITLTNR			1	47-48	NatC/E type
NU116/NUP116/YMR047C	MFGVSR			1	100	NatC/E type
TMA20/YER007C-A	MFKKFTR			1	97	NatC/E type
SRO77/YBL106C	MFKKSR			1	100	NatC/E type
DPOE/POL2/YNL262W	MFGKKKNNGGSSTAR	M2	F3	2	100	NatC/E type
RSF2/YJR127C	MFVNGNQSNFAKPAGQG…	M116	F116	116	76	NatC/E type

SSN3/YPL042C	MYNGKDR	M	Y	1	81	New NatC
MCA1/YOR197W	MYPGSGR			1	99	New NatC
NU157/NUP157/YER105C	MYSTPLKKR			1	97-100	New NatC
EKI1/YDR147W	MYTNYSLTSSDAMPR			1	44-66	New NatC
RIR1/YER070W	MYVYKR			1	67-85	New NatC
POF1/YCL047C	MKKTFEQFR	M	K	1	13-29	New NatC
ITR2/YOL103W	MKNSTAASSR			1	11-17	New NatC
TOM70/YNL121C	MKSFITR			1	9-21	New NatC
MG101/MGM101/YJR144W	MKSIFKVR			1	100	New NatC
ATG8/YBL078C	MKSTFKSEYPFEKR			1	16-18	New NatC
SCM3/YDL139C	MKTNKKISKR			1	90-100	New NatC
OTU2/YHL013C	MKKQATKSKR	M34	K35	34	96	New NatC

DAD1/YDR016C	MMASTSNDEEKLISTTDKYFIEQR	M	M	1	20	New NatC
LOT5/YKL183W	MMKKKPKCQIAR			1	44-53	New NatC
NGR1/YBR212W	MMSNVANASQR			1	94-96	New NatC
HOT1/YMR172W	MMPTTLKDGYR	M37	M38	37	100	New NatC

ENO1/YGR254W	MAVSKVYAR	M	A	1	0-5	New NatC

RL44A/RPL42A/YNL162W	MVNVPKTR	M	V	1	5-8	New NatC

CYPH/CPR1/YDR155C	MSQVYFDVEADGQPIGR	M	S	1	13	New NatC

Unique protein N-termini identified by N-terminal COFRADIC as being (partially) Nt-acetylated in WT yeast, while non–Nt-acetylated in *naa30*Δ yeast. Note that P1′ and P2′ (third and fourth column) refers to peptide positions and that these do not represent the protein positions for proteins indicated with a start > position 2 (fifth column).

In addition to the yNatC substrate N-termini that were Nt-free in y*naa30*Δ, we also identified N-termini that had a minimum reduction of 10% in Nt-acetylation degree when comparing *naa30*Δ to WT yeast but still had residual Nt-acetylation in *naa30*Δ yeast (≥5%). One might hypothesize that such N-termini may rely on both NatC as well as (a) redundant yeast NAT(s) for Nt-acetylation. These proteins held a variety of N-termini, including canonical NatC substrates (MF and MI) as well as others (MY, MN, ME, MM, MV, S, A) (Table S1). While such data do not represent firm evidence of NatC-mediated Nt-acetylation, they suggest a level of redundancy, implying that another yeast NAT may have the capacity to Nt-acetylate presumed NatC substrates such as MI, MF, and MY N-termini. Seventeen proteins had the same Nt-acetylation status in the WT and *naa30*Δ yeast (Table S1) despite belonging to the here-defined NatC category of N-termini (Table 1). For example, the MY N-terminus of Tgl1 is approximately 50% Nt-acetylated both in WT yeast and in *naa30*Δ yeast. However, most of these 17 N-termini belong to classes for which the responsible NAT is not properly determined such as MK, MH, and also a number of MG, MS, MT, and MV N-termini (Table S1). Thus, compensation between Naa30 and Naa50, previously shown to display similar N-terminal preferences (79), should likely also be considered in yeast.

**Figure 3:**
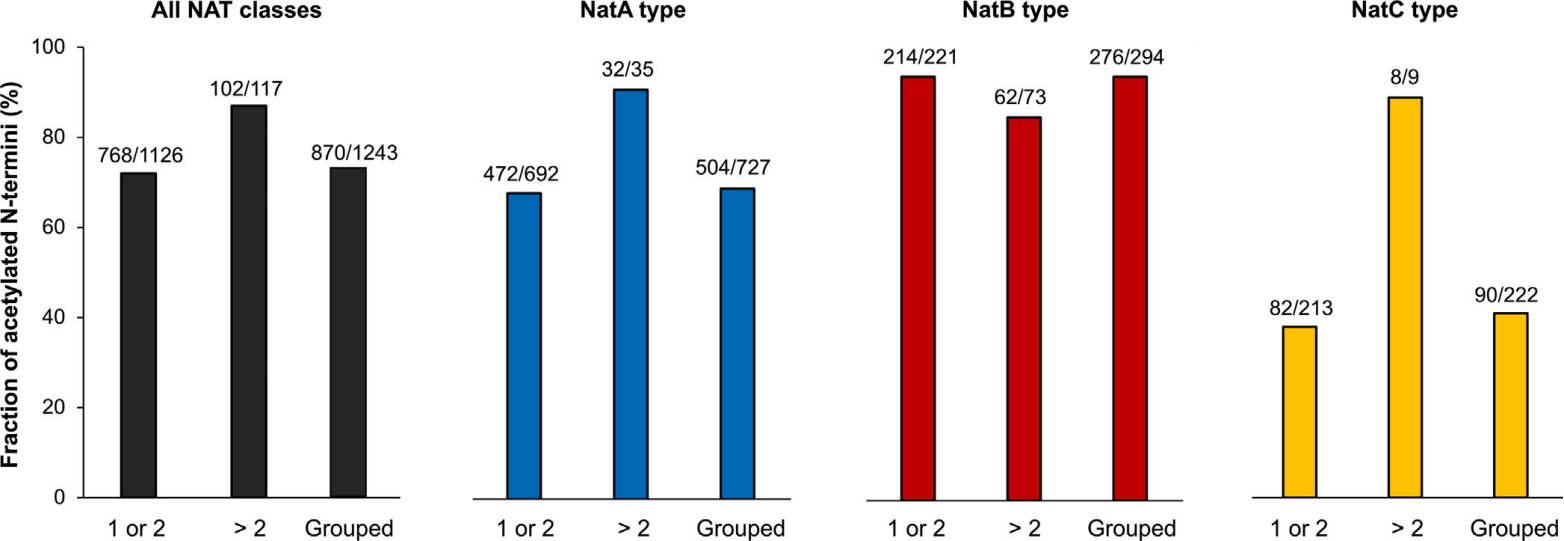
**Among proteins with an N-terminus of the NatC-type category, proteins with analternative,internal N-terminus are more frequently Nt-acetylated than proteins with a canonical N-terminus.** The protein N-termini detected in this dataset were divided into two groups according to their N-terminal start position. Either starting at position 1 or 2 (labeled 1 or 2) or after amino acid position 2 (labeled >2), within each NAT substrate class, the fraction of Nt-acetylated N-termini was calculated. NAT, N-terminal acetyltransferase.

Interestingly, our N-terminome analysis also revealed a difference between N-termini starting on position 1 or 2 and those with an alternative or internal start position >2 (Fig. 3). Comparing these two categories, irrespective of NAT substrate class categorization, the “start >2”-group of N-termini were more frequently Nt-acetylated (87%) than the “start 1 or 2”-group N-termini (72%, excluding MP). Interestingly, within the NatC category, the degree of Nt-acetylation was much higher for the “start >2”-group (89% vs 38% Nt-acetylated). Opposingly, among the NatB categorized N-termini, the Nt-acetylation degree of “start >2”-group N-termini is somewhat lower than that of the “start 1 or 2” group (85% vs 94%), whereas the NatA class follows the same trend as the NatC category (68% vs 91%) (Fig. 3). Thus, NatC seems to be particularly active towards proteins whose N-termini start after the annotated protein position 2 (in addition to matching the sequence preference).

**Figure 4:**
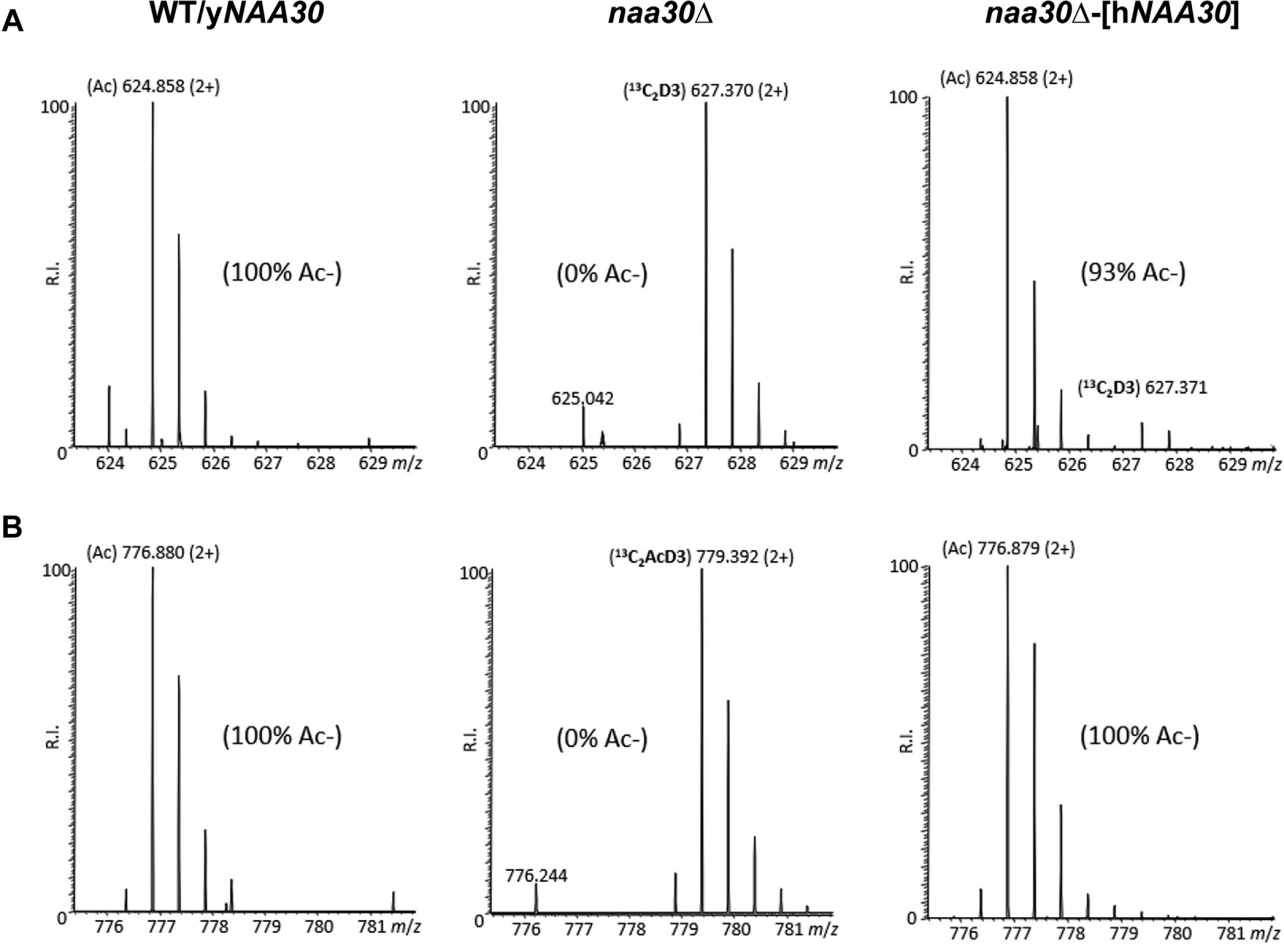
**Human NAA30 can (partially) restore yeast NatC substrate Nt-acetylation.** Representative MS-spectra of two yeast NatC substrates (*A*) partially (SSC1 (ML-), P0CCS90) or (*B*) fully (YDR239C (MF-), Q03780) restored in their Nt-acetylation status by ectopic expression of h*NAA30* in *naa30*Δ yeast. NAT, N-terminal acetyltransferase.

Our phenotypic data indicating that the human catalytic subunit NAA30 could rescue both *Nfs* ^*–*^ and Arl3 mislocalization phenotypes, suggested an evolutionary relationship between yeast and human NAA30. Hence, we hypothesized that hNAA30 could compensate for the loss of yNaa30 substrate Nt-acetylation upon ectopic expression in y*naa30*Δ yeast. A comparative analysis of WT *versus naa30*Δ or *naa30*Δ-[h*NAA30*] yeast by N-terminomics indeed demonstrated that hNAA30 is active over yeast NatC substrates (Tables S1 and S2). Representative MS spectra of three yeast NatC substrates fully or partially restored in their Nt-acetylation status in *naa30*Δ-[h*NAA30*] yeast are shown in Figure 4. Several of the here identified NatC substrate N-termini were Nt-acetylated to a similar degree in the yNaa30/WT and *naa30*Δ-[h*NAA30*] strains (25 out of the 57 NatC substrates with Nt-acetylation status restored to at least 67% of its level in WT yeast). However, many NatC substrates were less Nt-acetylated by hNAA30 compared to yNaa30 (5/57 substrates with 33–67% restored NtAc; 13/57 with 2–33%, and 8/57 with 0% restoration) (Tables S1 and S2). In particular, we observed a poor rescue capacity of hNAA30 towards MK-starting N-termini, as all proteins in this group (7) were only 0 to 22% restored in their Nt-acetylation status (Tables S1 and S2).

In *naa30*Δ yeast expressing hNAA30, we also found cases of N-termini with an increase in Nt-acetylation. This concerns both presumed non-yNatC substrates within the NatC/E/other substrate category as well as few NatB-type N-termini. Examples are POP6 (MI N-terminus) and KAPC (MY N-terminus) which are Nt-free in both WT and *naa30*Δ yeast, while 3 to 6% Nt-acetylated in the hNAA30-expressing strain; and FKH2 and BNI1 with NatB-type N-termini (ME-starting) detected as less than 10% Nt-acetylated in both WT and *naa30*Δ yeast, while over 60% Nt-acetylated in the hNAA30-expressing strain (Table S1).

## Discussion

### NAA30 orthologous complementation in NatC function

Here, we identified more than 50 unique yNatC substrates and showed that hNAA30 was able to rescue phenotypes and largely restore yNaa30 substrate Nt-acetylation in *naa30*Δ yeast. With about half of the yNatC substrates restored to similar Nt-acetylation levels as in WT yeast, we conclude that there is a strong conservation in substrate specificity between yeast and human NAA30.

In general, hNAA30 Nt-acetylates canonical NatC substrates to a lesser degree than yNaa30, while several non-yNatC substrates were additionally acetylated by hNAA30, possibly reflecting subtle differences in the *in vivo* substrate preferences of NatC/NAA30 in the two species. This is reminiscent to what has been observed for NatA (1) and NatB (50). However, for these two NATs, only subunits from the same species were able to function together. For NatC, the altered activity may therefore also be a result of suboptimal heterologous complex formation. Another yeast complementation study suggested that ectopically expressed hNAA30 indeed participates in a heterologous complex with yeast auxiliary subunits (66). In *Arabidopsis thaliana,* phenotypic analysis indicated that in plants, NAA30 function does not depend on NatC complex formation (83). While NAA30 performs catalysis and NAA35 is likely to mediate ribosome association, less is known about the function of NAA38. Nevertheless, the obligatory role of all three subunits for NatC-mediated Nt-acetylation, established in the early phenotyping, was recently revealed at the molecular level for the *S. cerevisiae* (60) and *S. pombe* (59) ternary complex, making future research on the function of the third subunit exciting.

Importantly, expression of human NAA30 was sufficient as a rescue model, without the need for a complete human complex. Thus, the current yeast model may become useful in future studies investigating the functionality of any human pathological variants of NAA30, by assessing Arl3 localization or growth in glycerol-containing medium. Regarding the latter, a high-throughput assay using a plate reader to monitor yeast growth has been reported (77).

### Biological role of NatC via its substrates

Importantly, our N-terminomics analyses uncovered several new NatC substrates that are of interest for functional follow-up studies. Experimental evidence of Nt-acetylation is deemed required to univocally assign NatC substrates due to generally lower degree of Nt-acetylation. Several previously studied, at the time putative/predicted, NatC substrates Rfc2, Rrn11, Tma20, Lrg1, Sly41, Bem1, Gshr (Glr1), and Nu157 (63) were now confirmed as yeast NatC substrates. These candidates were selected based on their detection as Nt-acetylated in other proteomics datasets as well as fitting to the NatC preference known at the time. However, none of these NatC substrates were presenting any gross changes in protein localization upon NatC perturbation (63).

Gene ontology–term analysis of the 57 yNatC substrates indicated enrichment of proteins involved in metabolism, pseudo hyphal growth, and oxidative stress response (Fig. S1). Interesting follow-up candidates could be Nfs1, Isf1, Sry1, Pdc2, Ssn3, Rir1, Msn4, and Hot1 (Table 1). Hot1 is a transcription factor that is required for the transient induction of glycerol biosynthetic genes *GPD1* and *GPP2* in response to high osmolarity (84). Intriguingly, the here identified NatC substrate Sly41 (Table 1), as well as the BioGRID Naa30/Naa35 interaction partner Dun1, next to Naa30 were identified as targets when screening for yeast deletion strains that grow poorly under anoxic conditions (68). Such cases of shared phenotypes could be worthy of further investigations. Moreover, gene ontology terms related to mitochondrial function and localization were enriched among yNatC substrates. Herein, Ngr1, Hot13, Hsp77, Gshr (Glr1), Pof1 and Tom70, Odpa (Pda1), and Etr1 (Table 1) could be good candidates for future protein localization, stabilization, and functional assays. Thus, in terms of explaining the *Nfs* ^*–*^ phenotype, several pieces have been added to the puzzle, although additional analyses are needed to define if and how these pieces come together to manifest *naa30*Δ yeast phenotypes.

Nt-acetylation has recently been suggested to be under regulatory control (33, 34, 35). Regarding NatC, an interesting hypothesis is that upstream regulation could facilitate environmental or metabolic adaptation as some research points in this direction: In yeast, the expression of *NAA35* is repressed in the presence of glucose and highly elevated in case of glycerol (67). In *Caenorhabditis elegans*, under rich environmental conditions, NatC negatively impacts on stress resistance and entry to the dormant larvae dauer stage, whereas in scarcity, NatC downregulation cause increased resistance to several stressors, including oxidative stress (85).

### Substrate specificity of *S. cerevisiae* NatC

In addition to the previously defined *S. cerevisiae* NatC-type N-termini, we here established several novel types of N-termini as yNatC substrates, including MY, MK, MM, MA, MV, and MS N-termini. This is in good agreement with the recently extended substrate profile of hNAA30/hNatC which includes ML, MI, MF, MW, MV, MM, MH, and MK N-termini (73). Our data further revealed an unanticipated complexity of NAT contributions to the yeast Nt-acetylome. First, for several yeast proteins including some MF, MI, MY, MM, MV, MN, and ME N-termini, we observed that both NatC and (an)other yeast NAT(s) contribute to their physiological Nt-Ac levels (Table S1). For MN and ME, the other yeast NAT is very likely NatB as these N-terminal sequences are mostly Nt-acetylated by NatB (50). Second, we found several proteins harboring non-NatB type M-starting N-terminal sequences to be exclusively acetylated by yeast NAT(s) other than NatC: MY, MK, MH, MG, MS, MT, and MV. Based on their established substrate specificity, it is not very likely that these N-termini are targeted by NatA or NatB. However, one MH-starting protein, Gch1, was found in one of the soluble *naa30*Δ fractions to be 20% Nt-acetylated (Table S1) and, previously, in a total lysate analysis of WT and yNatBΔ yeast, this protein was found to shift from 20% to 0% Nt-Ac (50). Thus, in this case, it seems likely that NatB may be the responsible NAT for the partial Nt-acetylation of an MH N-terminus. However, in other cases, it is clear that NatB is not responsible for Nt-acetylation of yeast MH N-termini, such as for Lsm7 and Ypo22, both found to be equally Nt-acetylated in WT *versus* NatBΔ strains (50).

Besides the three major yeast NATs already discussed and extensively profiled via N-terminal proteomics, NatA-NatC, there is also yeast Naa40/NatD acetylating histones H2A, H2A.Z, H4, as well as a few more SG-starting proteins (86, 87, 88). Its stringent specificity makes NatD a very unlikely candidate for acetylating M-starting N-termini. However, Naa50/NatE (89, 90) displays NAT-activity towards a variety of iM-starting N-termini *in vitro* and *in vivo,* including ML, MK, MM, MY, MV, and MS (78, 79, 91). However, in contrast to human NAA50, *S. pombe* and *S. cerevisiae* Naa50 were found to be inactive *in vitro* questioning their function as true NATs in these species (92). Further, *in vivo* data in *S. cerevisiae naa50*Δ were inconclusive with respect to endogenous NAT substrates of yNaa50 as no substrates could be identified (79). One such potential candidate substrate of Naa50 reported in this study was the MS-starting Pyruvate decarboxylase isozyme 1 (Pdc1), which was non-Nt-acetylated in *naa50*Δ, while its degree of Nt-acetylation could not be defined in WT yeast. In the total lysate samples of WT and *naa30*Δ yeast of the current dataset, however, Pdc1 was found to be 14% and 11% Nt-acetylated, respectively (Table S1), meaning that Pdc1 could be *in vivo* Nt-acetylated by *S. cerevisiae* Naa50/NatE and not Naa30/NatC.

**Figure 5:**
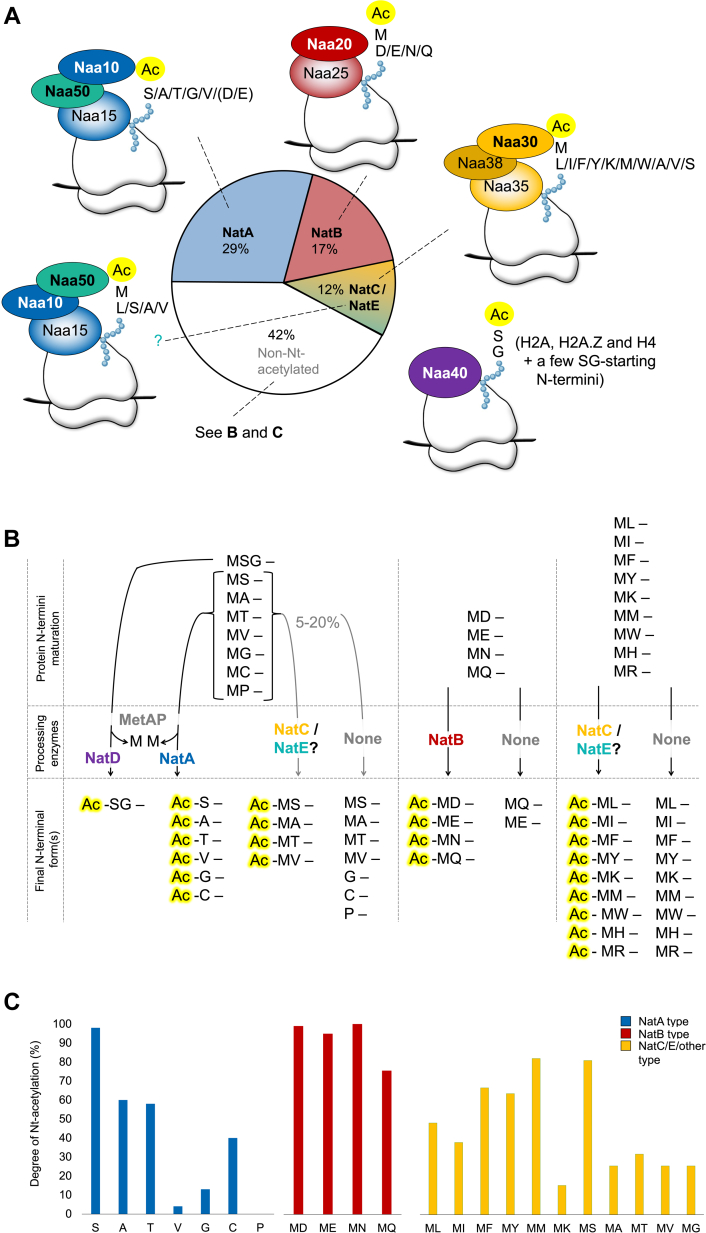
**N-terminal substrate specificities of *Saccharomyces cerevisiae* NATs and their proteome coverage.***A*, overview of the *S. cerevisiae* N-terminal acetyltransferases and their preferred N-terminal substrate specificities and proteome coverage. Yeast NatC is known to Nt-acetylate ML-, MI-, and MF- and MW-starting N-termini, and based on the results presented in the current study, also MY-, MK-, and MM- in addition to some MA-, MV- and MS-starting N-termini. NatD/Naa40 specifically Nt-acetylates histones H2A, H2A.Z, and H4 in addition to a few other SG-starting protein and is therefore not shown in the pie chart. Naa50 may associate with the NatA subunits to form NatE. *B*, fates of three classes of protein N-termini. Additional N-termini can be considered putative NatC substrates based on the types of N-termini revealed by the current N-terminome analysis, not only to cover additional M-«hydrophobic/amphipathic» N-termini within the NatC/E/other substrate class but also some non-MetAP–processed M-«small» type N-termini. *C*, estimated tendency for the indicated N-terminus types to be targeted by Nt-acetylation. For example, 23/31 (74%) of the here detected MQ-starting N-termini were NAT substrates. The size of the Nt-acetylome as shown in A is estimated by combining the % Nt-acetylation coverage (shown in in *C*) with the proteome-abundance of the N-terminus type. MetAP, methionine aminopeptidase; NAT, N-terminal acetyltransferase.

We summarize the current knowledge on *S. cerevisiae* NAT substrate specificities in Figure 5, now additionally including our proteome-wide findings on the uncovered yNatC substrate repertoire reported in this study. Compared to NatA and NatB substrates, which seem to be easily predictable, only half of the NatC substrates are Nt-acetylated and typically to a lower degree than NatA and NatB substrates. This suggests the existence of additional major determinants that steer whether a particular ML/MI/MF/MY/MK/MM/MV/MS/MA N-terminus is acetylated or not.

This study thus provides not only identification of concrete yNatC substrates but also facilitates future predictions of NatC substrates. This may facilitate further understanding of the molecular mechanisms of NatC-specific phenotypes, including the understanding of any environmentally responsive biological processes potentially regulated by NatC.

## Experimental procedures

### Yeast strains, plasmids, and cultivation

A complete list of all strains used in this study can be found in Table S3. All strains are derivatives of BY4742 (*MAT***α** *his3Δ1; leu2Δ0; lys2Δ0; ura3Δ0*) or BY4741 (*MATa; his3Δ1; leu2Δ0; met15Δ0; ura3Δ0).* Strain BY4742; YPR051wΔ::*kanMX4* (Acc: Y15470) was obtained from the yeast deletion collection (EUROSCARF) and used as a source for the YPR051wΔ::*kanMX4* cassette for the creation of a PCR-based substrate for homologous recombination to construct *naa30Δ* strains as reported previously (63, 66). BY4741*; ARL3::GFP-HIS3MX6* was obtained from the yeast GFP collection (Invitrogen/Life). WT and *naa30Δ* strains of BY4742 and BY4741 were transformed with the plasmid pBEVY-U. In addition, the *naa30Δ* strain was transformed with plasmids for expression of yeast *NAA30* (pBEVY-U-y*NAA30*) or human *NAA30* (pBEVY-U-HA-h*NAA30*). All *NAA* coding sequences and additional Kozak motifs and the HA-tag (if any) were inserted into the pBEVY-U downstream of the *ADH1* promoter using restriction *XmaI* and *EcoRI* cloning. All yeast cultivation was done in rich YPD or synthetic SC-Ura medium (0.67% Yeast nitrogen base without amino acids and ammonium sulfate, 0.17% Yeast synthetic drop-out medium supplements without uracil, 0.83% ammonium sulfate, and 3.33% glucose). Nfs-growth assays were performed using SC-Ura medium in which glycerol (3%) replaced glucose.

### N-terminomics (COFRADIC) sample preparation and analysis

Total lysates from WT, *naa30*Δ, and *naa30*Δ-[h*NAA30*] strains were collected at exponential growth phase and subjected to glass bead lysis as described previously (1). Additionally, subcellular fractionation was performed on all three strains to improve proteome coverage and to enrich different subcellular compartments. This was performed according to established protocols (93, 94), with some minor adjustments. First, the yeast cell wall was removed by resuspension of the yeast pellets in 40 mM β-mercaptoethanol and 0.25 mg/ml Zymolyase 100T-containing spheroplast buffer (1.4 M sorbitol, 50 mM potassium phosphate KPi [pH 7.5]). The resulting spheroplasts were dissolved in resuspension buffer (0.8 M sorbitol, 1 mM EDTA, 10 mM triethanolamine [titrated to pH 7.2 with 80% acetic acid], supplemented with 1 tablet Roche complete EDTA-free protease inhibitor per 100 ml) and lysed by repetitive pipetting (10x) through a 10-μl pipette tip attached to a 1-ml pipette tip and incubation on ice for 15 min followed by fractionation by successive differential centrifugation at 4 °C with increasing speed and duration (300*g* for 4 min; 2000*g* for 10 min; and 100,000*g* for 1 h 30 min). Material from three compartments were saved for combined fractional diagonal chromatography: (*i*) low-speed pelleted organelle-enriched fraction (P (pellet) 2000*g*), (*ii*) high-speed pelleted organelle enriched fraction (P 100,000*g*), and (*iii*) cytosolic fraction (S (soluble fraction) 100,000*g*). During further sample processing, P 2000*g* and P 100,000*g* were each divided into a soluble and less-soluble fraction that were ran separately, as performed previously (41). Samples were subjected to N-terminal combined fractional diagonal chromatography analysis to identify Nt-acetylated N-termini and quantify their degree of Nt-acetylation as described previously (2, 50). A difference of 10% in the degree of Nt-acetylation was defined as the minimum difference for defining a shift in the degree of Nt-acetylation, with the exception of the 5% minimum differences considered in case of the clear absence of the isotopic envelope matching either the fully (100%) Nt-Ac N-terminus or completely free (0%) Nt-Ac N-terminus form in one of the setups (2).

### Yeast growth assay

Over-night cultures of two independent clones per genotype were diluted to A_600_ 0.05 and grown at 30 °C and 250 rpm in three technical parallel cultures of either normal SC-Ura containing glucose or the equivalent SC-Ura glycerol in which glycerol (3%) replaced glucose. A_600_ was measured at intervals of approximately 6 h for a 62-h period.

### Yeast imaging

For imaging of live GFP yeast, strains were diluted to A_600_ 0.05 from over-night cultures and allowed to grow for 4 h until early exponential phase (A_600_ 1 ± 0.2). At this point, cells were washed three times in PBS containing 3% glucose and dissolved in the same buffer. A 2-μl drop of cell suspension was placed between an objective glass and coverslip. Imaging was performed as previously described (63). A series of random fields of view were imaged in order to retrieve localization information from at least 100 cells per genotype.

## Data availability

All processed data are contained within the article and Supplemental Information files. RAW files of the proteomic sets from the 18 samples analyzed here are available in PRIDE (accession number pending).

## Supporting information

This article contains supporting information.

## Conflict of interest

The authors declare that they have no conflicts of interest with the contents of this article.

## References

[1] Arnesen T., Van Damme P., Polevoda B., Helsens K., Evjenth R., Colaert N. (2009). Proteomics analyses reveal the evolutionary conservation and divergence of N-terminal acetyltransferases from yeast and humans. Proc. Natl. Acad. Sci. U. S. A..

[2] Van Damme P., Hole K., Pimenta-Marques A., Helsens K., Vandekerckhove J., Martinho R.G. (2011). NatF contributes to an evolutionary shift in protein N-terminal acetylation and is important for normal chromosome segregation. PLoS Genet..

[3] Aksnes H., Hole K., Arnesen T. (2015). Molecular, cellular, and physiological significance of N-terminal acetylation. Int. Rev. Cell Mol. Biol..

[4] Aksnes H., Drazic A., Marie M., Arnesen T. (2016). First things first: vital protein marks by N-terminal acetyltransferases. Trends Biochem. Sci..

[5] Bienvenut W.V., Sumpton D., Martinez A., Lilla S., Espagne C., Meinnel T. (2012). Comparative large scale characterization of plant *versus* mammal proteins reveals similar and idiosyncratic N-alpha-acetylation features. Mol. Cell Proteomics.

[6] Rope A.F., Wang K., Evjenth R., Xing J., Johnston J.J., Swensen J.J. (2011). Using VAAST to identify an X-linked disorder resulting in lethality in male infants due to N-terminal acetyltransferase deficiency. Am. J. Hum. Genet..

[7] Kalvik T.V., Arnesen T. (2013). Protein N-terminal acetyltransferases in cancer. Oncogene.

[8] McTiernan N., Tranebjaerg L., Bjorheim A.S., Hogue J.S., Wilson W.G., Schmidt B. (2022). Biochemical analysis of novel NAA10 variants suggests distinct pathogenic mechanisms involving impaired protein N-terminal acetylation. Hum Genet..

[9] Morrison J., Altuwaijri N.K., Bronstad K., Aksnes H., Alsaif H.S., Evans A. (2021). Missense NAA20 variants impairing the NatB protein N-terminal acetyltransferase cause autosomal recessive developmental delay, intellectual disability, and microcephaly. Genet. Med..

[10] Myklebust L.M., Van Damme P., Stove S.I., Dorfel M.J., Abboud A., Kalvik T.V. (2015). Biochemical and cellular analysis of Ogden syndrome reveals downstream Nt-acetylation defects. Hum. Mol. Genet..

[11] Park S.E., Kim J.M., Seok O.H., Cho H., Wadas B., Kim S.Y. (2015). Control of mammalian G protein signaling by N-terminal acetylation and the N-end rule pathway. Science.

[12] Muffels I.J.J., Wiame E., Fuchs S.A., Massink M.P.G., Rehmann H., Musch J.L.I. (2021). NAA80 bi-allelic missense variants result in high-frequency hearing loss, muscle weakness and developmental delay. Brain Commun..

[13] Scott D.C., Monda J.K., Bennett E.J., Harper J.W., Schulman B.A. (2011). N-terminal acetylation acts as an avidity enhancer within an interconnected multiprotein complex. Science.

[14] Monda J.K., Scott D.C., Miller D.J., Lydeard J., King D., Harper J.W. (2013). Structural conservation of distinctive N-terminal acetylation-dependent interactions across a family of mammalian NEDD8 ligation enzymes. Structure.

[15] Scott D.C., Hammill J.T., Min J., Rhee D.Y., Connelly M., Sviderskiy V.O. (2017). Blocking an N-terminal acetylation-dependent protein interaction inhibits an E3 ligase. Nat. Chem. Biol..

[16] Arnaudo N., Fernandez I.S., McLaughlin S.H., Peak-Chew S.Y., Rhodes D., Martino F. (2013). The N-terminal acetylation of Sir3 stabilizes its binding to the nucleosome core particle. Nat. Struct. Mol. Biol..

[17] Yang D., Fang Q., Wang M., Ren R., Wang H., He M. (2013). Nalpha-acetylated Sir3 stabilizes the conformation of a nucleosome-binding loop in the BAH domain. Nat. Struct. Mol. Biol..

[18] Behnia R., Panic B., Whyte J.R., Munro S. (2004). Targeting of the Arf-like GTPase Arl3p to the Golgi requires N-terminal acetylation and the membrane protein Sys1p. Nat. Cell Biol..

[19] Setty S.R., Strochlic T.I., Tong A.H., Boone C., Burd C.G. (2004). Golgi targeting of ARF-like GTPase Arl3p requires its Nalpha-acetylation and the integral membrane protein Sys1p. Nat. Cell Biol..

[20] Dikiy I., Eliezer D. (2014). N-terminal acetylation stabilizes N-terminal helicity in lipid- and micelle-bound alpha-synuclein and increases its affinity for physiological membranes. J. Biol. Chem..

[21] Miotto M.C., Valiente-Gabioud A.A., Rossetti G., Zweckstetter M., Carloni P., Selenko P. (2015). Copper binding to the N-terminally acetylated, naturally occurring form of alpha-synuclein induces local helical folding. J. Am. Chem. Soc..

[22] Forte G.M., Pool M.R., Stirling C.J. (2011). N-terminal acetylation inhibits protein targeting to the endoplasmic reticulum. PLoS Biol..

[23] Hwang C.S., Shemorry A., Varshavsky A. (2010). N-terminal acetylation of cellular proteins creates specific degradation signals. Science.

[24] Shemorry A., Hwang C.S., Varshavsky A. (2013). Control of protein quality and stoichiometries by N-terminal acetylation and the N-end rule pathway. Mol. Cell.

[25] Varshavsky A. (2019). N-degron and C-degron pathways of protein degradation. Proc. Natl. Acad. Sci. U. S. A..

[26] Kats I., Khmelinskii A., Kschonsak M., Huber F., Kniess R.A., Bartosik A. (2018). Mapping degradation signals and pathways in a eukaryotic N-terminome. Mol. Cell.

[27] Kats I., Reinbold C., Kschonsak M., Khmelinskii A., Armbruster L., Ruppert T. (2022). Up-regulation of ubiquitin-proteasome activity upon loss of NatA-dependent N-terminal acetylation. Life Sci. Alliance.

[28] Vu T.T.M., Mitchell D.C., Gygi S.P., Varshavsky A. (2020). The Arg/N-degron pathway targets transcription factors and regulates specific genes. Proc. Natl. Acad. Sci. U. S. A..

[29] Mueller F., Friese A., Pathe C., da Silva R.C., Rodriguez K.B., Musacchio A. (2021). Overlap of NatA and IAP substrates implicates N-terminal acetylation in protein stabilization. Sci. Adv..

[30] Oh J.H., Hyun J.Y., Varshavsky A. (2017). Control of Hsp90 chaperone and its clients by N-terminal acetylation and the N-end rule pathway. Proc. Natl. Acad. Sci. U. S. A..

[31] Kang L., Moriarty G.M., Woods L.A., Ashcroft A.E., Radford S.E., Baum J. (2012). N-terminal acetylation of alpha-synuclein induces increased transient helical propensity and decreased aggregation rates in the intrinsically disordered monomer. Protein Sci..

[32] Holmes W.M., Mannakee B.K., Gutenkunst R.N., Serio T.R. (2014). Loss of amino-terminal acetylation suppresses a prion phenotype by modulating global protein folding. Nat. Commun..

[33] Linster E., Stephan I., Bienvenut W.V., Maple-Grodem J., Myklebust L.M., Huber M. (2015). Downregulation of N-terminal acetylation triggers ABA-mediated drought responses in Arabidopsis. Nat. Commun..

[34] Varland S., Aksnes H., Kryuchkov F., Impens F., Van Haver D., Jonckheere V. (2018). N-terminal acetylation levels are maintained during acetyl-CoA deficiency in Saccharomyces cerevisiae. Mol. Cell Proteomics.

[35] Molina-Serrano D., Schiza V., Demosthenous C., Stavrou E., Oppelt J., Kyriakou D. (2016). Loss of Nat4 and its associated histone H4 N-terminal acetylation mediates calorie restriction-induced longevity. EMBO Rep..

[36] Castrec B., Dian C., Ciccone S., Ebert C.L., Bienvenut W.V., Le Caer J.P. (2018). Structural and genomic decoding of human and plant myristoylomes reveals a definitive recognition pattern. Nat. Chem. Biol..

[37] Nevitt C., Tooley J.G., Schaner Tooley C.E. (2018). N-terminal acetylation and methylation differentially affect the function of MYL9. Biochem. J..

[38] Akimov V., Barrio-Hernandez I., Hansen S.V.F., Hallenborg P., Pedersen A.K., Bekker-Jensen D.B. (2018). UbiSite approach for comprehensive mapping of lysine and N-terminal ubiquitination sites. Nat. Struct. Mol. Biol..

[39] Bienvenut W.V., Brunje A., Boyer J.B., Muhlenbeck J.S., Bernal G., Lassowskat I. (2020). Dual lysine and N-terminal acetyltransferases reveal the complexity underpinning protein acetylation. Mol. Syst. Biol..

[40] Aksnes H., Ree R., Arnesen T. (2019). Co-translational, post-translational, and non-catalytic roles of N-terminal acetyltransferases. Mol. Cell.

[41] Aksnes H., Van Damme P., Goris M., Starheim K.K., Marie M., Stove S.I. (2015). An organellar Nα-acetyltransferase, Naa60, acetylates cytosolic N termini of transmembrane proteins and maintains Golgi integrity. Cell Rep..

[42] Linster E., Layer D., Bienvenut W.V., Dinh T.V., Weyer F.A., Leemhuis W. (2020). The Arabidopsis N(alpha) -acetyltransferase NAA60 locates to the plasma membrane and is vital for the high salt stress response. New Phytol..

[43] Dinh T.V., Bienvenut W.V., Linster E., Feldman-Salit A., Jung V.A., Meinnel T. (2015). Molecular identification and functional characterization of the first Nalpha-acetyltransferase in plastids by global acetylome profiling. Proteomics.

[44] Drazic A., Aksnes H., Marie M., Boczkowska M., Varland S., Timmerman E. (2018). NAA80 is actin's N-terminal acetyltransferase and regulates cytoskeleton assembly and cell motility. Proc. Natl. Acad. Sci. U. S. A..

[45] Starheim K.K., Gevaert K., Arnesen T. (2012). Protein N-terminal acetyltransferases: when the start matters. Trends Biochem. Sci..

[46] Mullen J.R., Kayne P.S., Moerschell R.P., Tsunasawa S., Gribskov M., Colavito-Shepanski M. (1989). Identification and characterization of genes and mutants for an N-terminal acetyltransferase from yeast. EMBO J..

[47] Polevoda B., Norbeck J., Takakura H., Blomberg A., Sherman F. (1999). Identification and specificities of N-terminal acetyltransferases from Saccharomyces cerevisiae. EMBO J..

[48] Moerschell R.P., Hosokawa Y., Tsunasawa S., Sherman F. (1990). The specificities of yeast methionine aminopeptidase and acetylation of amino-terminal methionine *in vivo*. Processing of altered iso-1-cytochromes c created by oligonucleotide transformation. J. Biol. Chem..

[49] Polevoda B., Cardillo T.S., Doyle T.C., Bedi G.S., Sherman F. (2003). Nat3p and Mdm20p are required for function of yeast NatB Nalpha-terminal acetyltransferase and of actin and tropomyosin. J. Biol. Chem..

[50] Van Damme P., Lasa M., Polevoda B., Gazquez C., Elosegui-Artola A., Kim D.S. (2012). N-terminal acetylome analyses and functional insights of the N-terminal acetyltransferase NatB. Proc. Natl. Acad. Sci. U. S. A..

[51] Tercero J.C., Wickner R.B. (1992). MAK3 encodes an N-acetyltransferase whose modification of the L-A gag NH2 terminus is necessary for virus particle assembly. J. Biol. Chem..

[52] Kimura Y., Takaoka M., Tanaka S., Sassa H., Tanaka K., Polevoda B. (2000). N(alpha)-acetylation and proteolytic activity of the yeast 20 S proteasome. J. Biol. Chem..

[53] Polevoda B., Sherman F. (2001). NatC Nalpha-terminal acetyltransferase of yeast contains three subunits, Mak3p, Mak10p, and Mak31p. J. Biol. Chem..

[54] Tercero J.C., Dinman J.D., Wickner R.B. (1993). Yeast MAK3 N-acetyltransferase recognizes the N-terminal four amino acids of the major coat protein (gag) of the L-A double-stranded RNA virus. J. Bacteriol..

[55] Wickner R.B. (1974). Chromosomal and nonchromosomal mutations affecting the "killer character" of Saccharomyces cerevisiae. Genetics.

[56] Wickner R.B., Leibowitz M.J. (1976). Chromosomal genes essential for replication of a double-stranded RNA plasmid of Saccharomyces cerevisiae: the killer character of yeast. J. Mol. Biol..

[57] Tercero J.C., Riles L.E., Wickner R.B. (1992). Localized mutagenesis and evidence for post-transcriptional regulation of MAK3. A putative N-acetyltransferase required for double-stranded RNA virus propagation in Saccharomyces cerevisiae. J. Biol. Chem..

[58] Starheim K.K., Gromyko D., Evjenth R., Ryningen A., Varhaug J.E., Lillehaug J.R. (2009). Knockdown of human N alpha-terminal acetyltransferase complex C leads to p53-dependent apoptosis and aberrant human Arl8b localization. Mol. Cell Biol..

[59] Deng S., Gottlieb L., Pan B., Supplee J., Wei X., Petersson E.J. (2021). Molecular mechanism of N-terminal acetylation by the ternary NatC complex. Structure.

[60] Grunwald S., Hopf L.V.M., Bock-Bierbaum T., Lally C.C.M., Spahn C.M.T., Daumke O. (2020). Divergent architecture of the heterotrimeric NatC complex explains N-terminal acetylation of cognate substrates. Nat. Commun..

[61] Liszczak G., Goldberg J.M., Foyn H., Petersson E.J., Arnesen T., Marmorstein R. (2013). Molecular basis for N-terminal acetylation by the heterodimeric NatA complex. Nat. Struct. Mol. Biol..

[62] Hong H., Cai Y., Zhang S., Ding H., Wang H., Han A. (2017). Molecular basis of substrate specific acetylation by N-terminal acetyltransferase NatB. Structure.

[63] Aksnes H., Osberg C., Arnesen T. (2013). N-terminal acetylation by NatC is not a general determinant for substrate subcellular localization in Saccharomyces cerevisiae. PLoS One.

[64] Behnia R., Barr F.A., Flanagan J.J., Barlowe C., Munro S. (2007). The yeast orthologue of GRASP65 forms a complex with a coiled-coil protein that contributes to ER to Golgi traffic. J. Cell Biol..

[65] Murthi A., Hopper A.K. (2005). Genome-wide screen for inner nuclear membrane protein targeting in Saccharomyces cerevisiae: roles for N-acetylation and an integral membrane protein. Genetics.

[66] Osberg C., Aksnes H., Ninzima S., Marie M., Arnesen T. (2016). Microscopy-based Saccharomyces cerevisiae complementation model reveals functional conservation and redundancy of N-terminal acetyltransferases. Sci. Rep..

[67] Lee Y.J., Wickner R.B. (1992). MAK10, a glucose-repressible gene necessary for replication of a dsRNA virus of Saccharomyces cerevisiae, has T cell receptor alpha-subunit motifs. Genetics.

[68] Samanfar B., Omidi K., Hooshyar M., Laliberte B., Alamgir M., Seal A.J. (2013). Large-scale investigation of oxygen response mutants in Saccharomyces cerevisiae. Mol. BioSystems.

[69] Sprague G.F., Cronan J.E. (1977). Isolation and characterization of Saccharomyces cerevisiae mutants defective in glycerol catabolism. J. Bacteriol..

[70] Grauslund M., Lopes J.M., Ronnow B. (1999). Expression of GUT1, which encodes glycerol kinase in Saccharomyces cerevisiae, is controlled by the positive regulators Adr1p, Ino2p and Ino4p and the negative regulator Opi1p in a carbon source-dependent fashion. Nucl. Acids Res..

[71] Grauslund M., Ronnow B. (2000). Carbon source-dependent transcriptional regulation of the mitochondrial glycerol-3-phosphate dehydrogenase gene, GUT2, from Saccharomyces cerevisiae. Can. J. Microbiol..

[72] Egner A., Jakobs S., Hell S.W. (2002). Fast 100-nm resolution three-dimensional microscope reveals structural plasticity of mitochondria in live yeast. Proc. Natl. Acad. Sci. U. S. A..

[73] Van Damme P., Kalvik T.V., Starheim K.K., Jonckheere V., Myklebust L.M., Menschaert G. (2016). A role for human N-alpha acetyltransferase 30 (Naa30) in maintaining mitochondrial integrity. Mol. Cell Proteomics.

[74] Stradalova V., Stahlschmidt W., Grossmann G., Blazikova M., Rachel R., Tanner W. (2009). Furrow-like invaginations of the yeast plasma membrane correspond to membrane compartment of Can1. J. Cell Sci..

[75] Polevoda B., Sherman F. (2000). Nalpha -terminal acetylation of eukaryotic proteins. J. Biol. Chem..

[76] van Deventer S., Menendez-Benito V., van Leeuwen F., Neefjes J. (2015). N-terminal acetylation and replicative age affect proteasome localization and cell fitness during aging. J. Cell Sci..

[77] Drazic A., Varland S. (2021). Human NAA30 can rescue yeast mak3 mutant growth phenotypes. Biosci. Rep..

[78] Van Damme P., Evjenth R., Foyn H., Demeyer K., De Bock P.J., Lillehaug J.R. (2011). Proteome-derived peptide libraries allow detailed analysis of the substrate specificities of N(alpha)-acetyltransferases and point to hNaa10p as the post-translational actin N(alpha)-acetyltransferase. Mol. Cell Proteomics.

[79] Van Damme P., Hole K., Gevaert K., Arnesen T. (2015). N-terminal acetylome analysis reveals the specificity of Naa50 (Nat5) and suggests a kinetic competition between N-terminal acetyltransferases and methionine aminopeptidases. Proteomics.

[80] Helsens K., Van Damme P., Degroeve S., Martens L., Arnesen T., Vandekerckhove J. (2011). Bioinformatics analysis of a Saccharomyces cerevisiae N-terminal proteome provides evidence of alternative translation initiation and post-translational N-terminal acetylation. J. Proteome Res..

[81] Goetze S., Qeli E., Mosimann C., Staes A., Gerrits B., Roschitzki B. (2009). Identification and functional characterization of N-terminally acetylated proteins in Drosophila melanogaster. PLoS Biol..

[82] Jonckheere V., Fijalkowska D., Van Damme P. (2018). Omics assisted N-terminal proteoform and protein expression profiling on methionine aminopeptidase 1 (MetAP1) deletion. Mol. Cell Proteomics.

[83] Pesaresi P., Gardner N.A., Masiero S., Dietzmann A., Eichacker L., Wickner R. (2003). Cytoplasmic N-terminal protein acetylation is required for efficient photosynthesis in Arabidopsis. Plant Cell.

[84] Rep M., Krantz M., Thevelein J.M., Hohmann S. (2000). The transcriptional response of Saccharomyces cerevisiae to osmotic shock. Hot1p and Msn2p/Msn4p are required for the induction of subsets of high osmolarity glycerol pathway-dependent genes. J. Biol. Chem..

[85] Warnhoff K., Murphy J.T., Kumar S., Schneider D.L., Peterson M., Hsu S. (2014). The DAF-16 FOXO transcription factor regulates natc-1 to modulate stress resistance in Caenorhabditis elegans, linking insulin/IGF-1 signaling to protein N-terminal acetylation. PLoS Genet..

[86] Song O.K., Wang X., Waterborg J.H., Sternglanz R. (2003). An Nalpha-acetyltransferase responsible for acetylation of the N-terminal residues of histones H4 and H2A. J. Biol. Chem..

[87] Hole K., Van Damme P., Dalva M., Aksnes H., Glomnes N., Varhaug J.E. (2011). The human N-alpha-acetyltransferase 40 (hNaa40p/hNatD) is conserved from yeast and N-terminally acetylates histones H2A and H4. PLoS One.

[88] Jonckheere V., Van Damme P. (2021). N-Terminal acetyltransferase Naa40p whereabouts put into N-terminal proteoform perspective. Int. J. Mol. Sci..

[89] Gautschi M., Just S., Mun A., Ross S., Rucknagel P., Dubaquie Y. (2003). The yeast N(alpha)-acetyltransferase NatA is quantitatively anchored to the ribosome and interacts with nascent polypeptides. Mol. Cell Biol..

[90] Arnesen T., Anderson D., Torsvik J., Halseth H.B., Varhaug J.E., Lillehaug J.R. (2006). Cloning and characterization of hNAT5/hSAN: An evolutionarily conserved component of the NatA protein N-alpha-acetyltransferase complex. Gene.

[91] Evjenth R., Hole K., Karlsen O.A., Ziegler M., Arnesen T., Lillehaug J.R. (2009). Human Naa50p (Nat5/San) displays both protein N alpha- and N epsilon-acetyltransferase activity. J. Biol. Chem..

[92] Deng S., Magin R.S., Wei X., Pan B., Petersson E.J., Marmorstein R. (2019). Structure and mechanism of acetylation by the N-terminal dual enzyme NatA/Naa50 complex. Structure.

[93] Rieder S.E., Emr S.D. (2001). Isolation of subcellular fractions from the yeast Saccharomyces cerevisiae. Curr. Protoc. Cell Biol..

[94] Rieder S.E., Emr S.D. (2001). Overview of subcellular fractionation procedures for the yeast Saccharomyces cerevisiae. Curr. Protoc. Cell Biol..

