# Supplementary material for: Expanded in vivo substrate profile of the yeast N-terminal acetyltransferase NatC
Source: J Biol Chem. 2022 Dec 22;299(2):102824. doi: 10.1016/j.jbc.2022.102824 (PMC9867985; doi:10.1016/j.jbc.2022.102824)
Supplement: Supporting Information [file mmc1.pdf]

Supporting Information

Table S1. (Separate file)
List of 1,596 yeast N-termini identified in the (subcellular) proteomes of WT, naa30∆, naa30∆[hNAA30] yeast. Identity of the amino acid residue (P1) preceding the identified N-terminus (if any), peptide sequence identified, identity of the first two amino acid residue (P1' and P2') of the identified N-terminus, start and end positions of identified N-termini, NAT type specificity, UniProt database primary accession number, UniProt entry name, gene name, corresponding protein description, N-terminal modification status/states confirmed by MS/MS (PSMs per modification/in total/per project), determined Nt-acetylation in % (per setup, strain, (subcellular) fraction) and overview of corresponding project numbers, strain identities and fractions are given. Further, the highest MASCOT score per N-terminal peptide is reported and whenever a peptide matched to multiple protein or multiple protein members of a protein family (redundancy), isoforms are given for all uniquely identified yeast N-termini. N-termini are grouped according to their UniProt database start position and compliance with the rules of N-terminal acetylation and iMet processing and ranked alphabetically according to their NAT type and sequence. Abbreviations used: TL: total lysates, Ace: Nt-acetylation, ($^{13}C_2D_3$)Ace: heavy/*in vitro* ($^{13}C_2$ and trideutero-)Nt-acetylation.

Table S2. *S. cerevisiae* NatC substrates identified and their degree of Nt-acetylation by hNAA30. Unique protein N-termini detected as Nt-acetylated in WT yeast and not in naa30Δ yeast with their Nt-acetylation status detected in the hNAA30 COFRADIC sample and estimated degree of Nt-acetylation rescue.

Protein (UNIPROT)	Nt-sequence	P1'	P2'	Start	NtAc in WT yeast	NtAc in naa30Δ[hNAA30]	Degree of rescue by hNAA30
HSP77	MLAAKNILNR	M	L	1	100 %	91-93 %	91-93 %
ODPA	MLAASFKR			1	97-100 %	87-92 %	91 %
PRP19	MLCAISGKVPR			1	64 %	not detected	N.D.
LST4	MLGNLLR			1	100 %	88-100 %	88-100 %
ADRX	MLKIVTR			1	100 %	17 %	17 %
BEM1	MLKNFKLSKR			1	100 %	95-100 %	95-100 %
NFS1	MLKSTATR			1	100 %	95-100 %	95-100 %
CRF1	MLLSAPVNSTVR			1	26 %	not detected	N.D.
YG1O	MLLTPAKTTR			1	54 %	0 %	0 %
HUL5	MLNFTGQTR			1	100 %	90-100 %	90-100 %
ETR1	MLPTFKR			1	100 %	96 %	96 %
YEY8	MLQQGSSSR			1	100 %	61-67 %	61-67 %
GSHR	MLSATKQTFR			1	100 %	93-96 %	93-96 %
NOT1	MLSATYR			1	100 %	82-84 %	82-84 %
SKS1	MLSDCLLNNFR			1	0-15 %	0 %	0 %
PDC2	MLSIQQR			1	10-15 %	0 %	0 %
BAP2	MLSSEDFGSSGKKETSPDSISIR			1	10 %	11 %	110 %
MSN4	MLVFGPNSSFVR			1	100 %	91-100 %	91-100 %
AIM18	MLKSLQR			9	80 %	9-11 %	12 %
LKHA4	MLPLSIEQR			40	7-29 %	0-10 %	22 %
PCD1	MLSSKQLIENLIR			8	58 %	5 %	9 %
AFG1	MIALKPNAVR	M	I	1	100 %	62 %	62 %
YJ133	MIAQSTR			1	8-20 %	3-25 %	83 %
ISF1	MIASEIFER			1	9-13%	not detected	N.D.
HOT13	MIETAIYGKTVDDQSR			1	1-8 %	0 %	0 %
LRG1	MIQNSAGYR			1	100 %	73-86 %	73-86 %
SLY41	MIQTQSTAIKR			1	94 %	46-58 %	82 %
ACM1	MISPSKKR			1	73-94 %	15-27 %	26 %
SRY1	MIVPTYGDVLDASNR			1	3-8 %	0 %	0 %
TFC6	MIKLR			246	100 %	75-92 %	75-92 %
YD239	MFDGFSNNKGKR	M	F	1	100 %	100 %	100 %
RFC2	MFEGFGPNKKR			1	99-100%	34-46 %	34-46 %
RRN11	MFEVPITLTNR			1	47-48%	0-3 %	2 %
DPOE	MFGKKKNNGGSSTAR			2	100 %	61-68 %	61-68 %
NU116	MFGVSR			1	100 %	97-100 %	97-100 %
TMA20	MFKKFTR			1	97 %	6-8 %	8 %
SRO77	MFKKSR			1	100 %	92-97 %	92-97 %
RSF2	MFVNGNQSNFAKPAGQGILPIPKKSR			116	76 %	not detected	N.D.
SSN3	MYNGKDR	M	Y	1	81 %	35 %	43 %
MCA1	MYPGSGR			1	99 %	88-93 %	91 %
NU157	MYSTPLKKR			1	97-100 %	50-87 %	67 %
EKI1	MYTNYSLTSSDAMPR			1	44-66 %	61-74 %	122 %
RIR1	MYVYKR			1	67-85 %	12-34 %	26 %
POF1	MKKTFEQFR	M	K	1	13-29 %	0 %	0 %
ITR2	MKNSTAASSR			1	11-17 %	0-5 %	15 %
TOM70	MKSFITR			1	9-21 %	0 %	0 %
MG101	MKSIFKVR			1	100 %	11-19 %	11-19 %
ATG8	MKSTFKSEYPFEKR			1	16-18 %	0-3 %	9 %
SCM3	MKTNKKISKR			1	90-100 %	7-11 %	8 %
OTU2	MKKQATKSKR			34	96 %	21 %	22 %
DAD1	MMASTSNDEEKLISTTDKYFIEQR	M	M	1	20 %	not detected	N.D.
LOT5	MMKKKPKCQIAR			1	44-53 %	8 %	17 %
NGR1	MMSNVANASQR			1	94-96%	68-79 %	78 %
HOT1	MMPTTLKDGYR			37	100 %	not detected	N.D.
ENO1	MAVSKVYAR	M	A	1	0-5%	0-4 %	88 %
RL44A	MVNVPKTR	M	V	1	5-8 %	0 %	0 %
CYPH	MSQVYFDVEADGQPIGR	M	S	1	13 %	6 %	47 %

Table S3. Yeast strains used in this study.

Strain	Short name	Genotype	Use
TA36	-	BY4742; *Matα; his3Δ1; leu2Δ0; lys2Δ0; ura3Δ0*	Strain generation
TA44	-	BY4742; *Matα; his3Δ1; leu2Δ0; lys2Δ0; ura3Δ0; naa30Δ::kanMX4*	Strain generation
TA47/135	Ctrl/WT/ yNaa30	BY4742; *Matα; his3Δ1; leu2Δ0; lys2Δ0; ura3Δ0; [pBEVY-U]*	Growth assay; COFRADIC (TA47)
TA61/62	*naa30Δ*	BY4742; *Matα; his3Δ1; leu2Δ0; lys2Δ0; ura3Δ0; naa30Δ::kanMX4; [pBEVY-U]*	Growth assay; COFRADIC (TA61)
TA124/125	*naa30Δ*- [h*NAA30*]	BY4742; *Matα; his3Δ1; leu2Δ0; lys2Δ0; ura3Δ0; naa30Δ::kanMX4; [pBEVY-U-HA-hNAA30]*	Growth assay; COFRADIC (TA124)
TA256	-	BY4741; *Mata; his3Δ1; leu2Δ0; met15Δ0; ura3Δ0; ARL3::GFP-HIS3MX6*	Strain generation
TA266	-	BY4741; *Mata; his3Δ1; leu2Δ0; met15Δ0; ura3Δ0; ARL3::GFP-HIS3MX6; naa30Δ::kanMX4*	Strain generation
TA371/372	Arl3-GFP, WT/Ctrl	BY4741; *Mata; his3Δ1; leu2Δ0; met15Δ0; ura3Δ0; ARL3::GFP-HIS3MX6; [pBEVY-U]*	Imaging
TA373/374	Arl3-GFP- *naa30Δ*	BY4741; *Mata; his3Δ1; leu2Δ0; met15Δ0; ura3Δ0; ARL3::GFP-HIS3MX6; naa30Δ::kanMX4; [pBEVY-U]*	Imaging
TA381/382	Arl3-GFP- *naa30Δ*,- [y*NAA30*]	BY4741; *Mata; his3Δ1; leu2Δ0; met15Δ0; ura3Δ0; ARL3::GFP-HIS3MX6; naa30Δ::kanMX4; [pBEVY-U-yNAA30]*	Imaging
TA375/376	Arl3-GFP- *naa30Δ*- [h*NAA30*]	BY4741; *Mata; his3Δ1; leu2Δ0; met15Δ0; ura3Δ0; ARL3::GFP-HIS3MX6; naa30Δ::kanMX4; [pBEVY-U-HA-hNAA30]*	Imaging

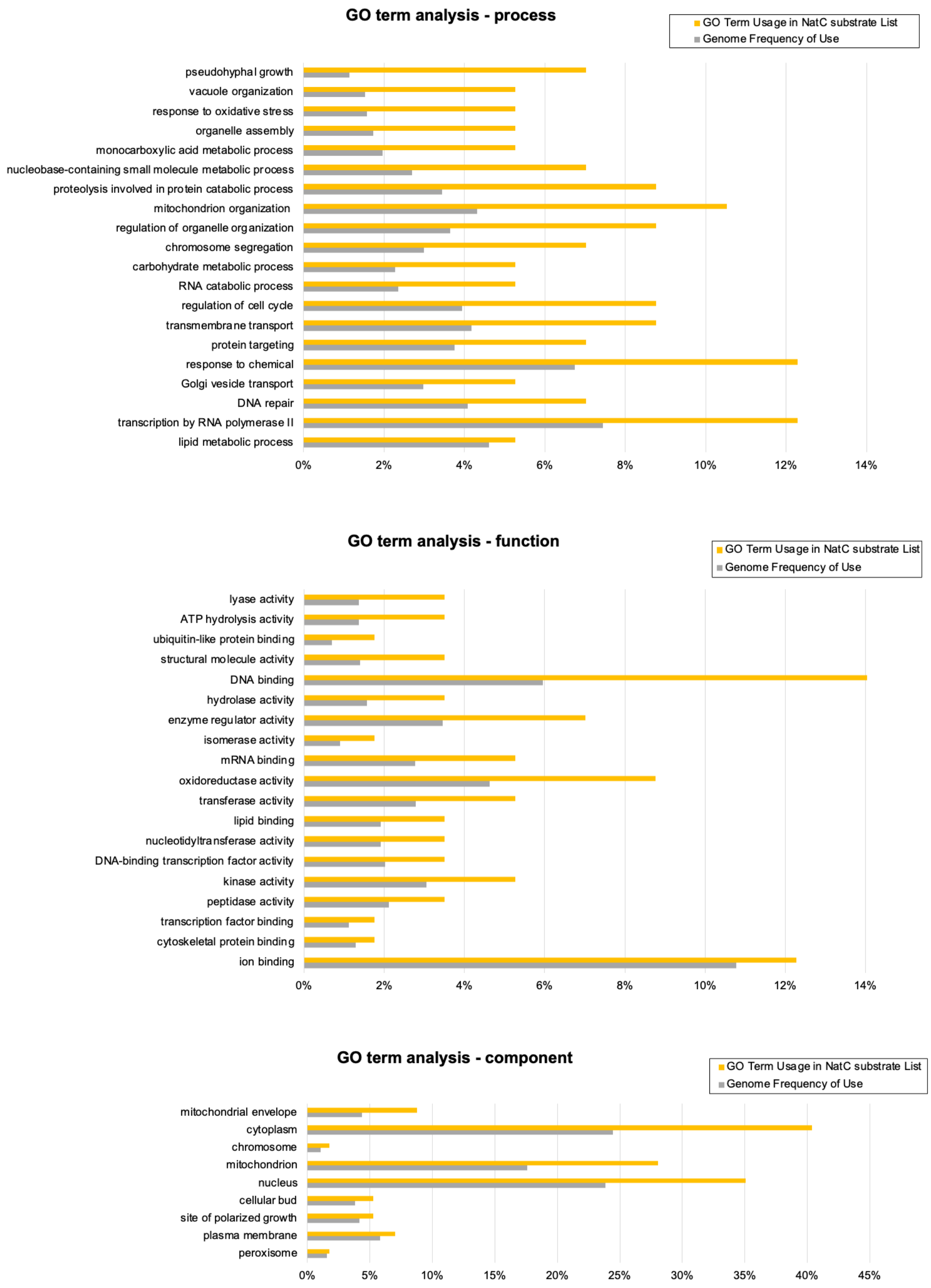

Figure S1. Yeast GO term analysis. The 57 proteins identified here as yeast NatC substrates were investigated for GO term frequency against GO term frequency in the *S. cerevisiae* genome (Slim GO at https://www.yeastgenome.org).
